# Rapid Expectation Adaptation during Syntactic Comprehension

**DOI:** 10.1371/journal.pone.0077661

**Published:** 2013-10-30

**Authors:** Alex B. Fine, T. Florian Jaeger, Thomas A. Farmer, Ting Qian

**Affiliations:** 1 Department of Brain and Cognitive Sciences, University of Rochester, Rochester, New York, United States of America; 2 Department of Computer Science, University of Rochester, Rochester, New York, United States of America; 3 Department of Psychology, University of Iowa, Iowa City, Iowa, United States of America; University of Leicester, United Kingdom

## Abstract

When we read or listen to language, we are faced with the challenge of inferring intended messages from noisy input. This challenge is exacerbated by considerable variability between and within speakers. Focusing on syntactic processing (parsing), we test the hypothesis that language comprehenders rapidly adapt to the syntactic statistics of novel linguistic environments (e.g., speakers or genres). Two self-paced reading experiments investigate changes in readers’ syntactic expectations based on repeated exposure to sentences with temporary syntactic ambiguities (so-called “garden path sentences”). These sentences typically lead to a clear expectation violation signature when the temporary ambiguity is resolved to an *a priori* less expected structure (e.g., based on the statistics of the lexical context). We find that comprehenders rapidly adapt their syntactic expectations to converge towards the local statistics of novel environments. Specifically, repeated exposure to *a priori* unexpected structures can reduce, and even completely undo, their processing disadvantage (Experiment 1). The opposite is also observed: *a priori* expected structures become less expected (even eliciting garden paths) in environments where they are hardly ever observed (Experiment 2). Our findings suggest that, when changes in syntactic statistics are to be expected (e.g., when entering a novel environment), comprehenders can rapidly adapt their expectations, thereby overcoming the processing disadvantage that mistaken expectations would otherwise cause. Our findings take a step towards unifying insights from research in expectation-based models of language processing, syntactic priming, and statistical learning.

## Introduction

Language use is *variable*, in the sense that each speaker of a language enters any given communicative situation with a unique set of life experiences that, put simply, shape how that person says things. For example, many people can relate to the experience of participating in a conversation in which both participants are native speakers of the language in use, but, based on differences in life experience, speak in different regional dialects, use different words for the same objects or concepts, and so forth. Although situations such as these can lead to confusions or misunderstandings, communication is typically successful. That is, with sufficient time, the systems responsible for real-time language comprehension seem to be able to accommodate considerable variability in the linguistic signal.

The fact that communication is generally successful is even more striking when we consider the full scope of linguistic variability beyond anecdotal experience with regional dialects and the like. Indeed, linguistic variability is the norm, not the exception, and it appears to be present at every level of linguistic representation. In speech perception research, variability presents an infamous problem known as the “lack of invariance” [1]: The same sound category (phoneme) in the same phonological context is realized differently even by speakers that, for all relevant purposes, would be classified as speaking the same dialect and sociolect [2]. Even productions by the same speaker differ over time [3]–[5] and can vary based on the social context, such as the register or speech style [6]. Speakers also differ in terms of their preferences for lexical items (e.g., *couch* vs. *sofa*) and syntactic structures (e.g., the choice between *Hand that man a banana* and *Hand a banana to that man*). For example, the frequency with which speakers of English produce the optional complementizer *that* (e.g., *Windom Earle said that he was coming to Twin Peaks*) varies based on dialect [7], genre [8], and modality [9]. Weiner and Labov (1983) [10] find that the choice between active (*Bob killed Laura Palmer*) and passive (*Laura Palmer was killed by Bob*) voice is influenced by the age, gender, and social status of the talker. Research in sociolinguistics is abundant with similar examples in which lexical, morphological, and syntactic preferences differ across geographical regions, time, modalities, and genres.

Variability is thus likely a general fact about the distribution of linguistic events in a population of speakers, thus raising the question of how the systems involved in real-time language processing address the challenges that result from linguistic variability. While the lack of invariance has long been recognized as one of the central problems that theories of speech perception need to address, the same problem has so far received relatively little attention in research on language understanding beyond speech perception, such as lexical and syntactic processing. Variability-based questions are particularly interesting in the context of accounts that emphasize the role of experience in language processing [11]–[13]. Such theories hold that comprehenders generate expectations–about the probability of observing particular sounds, words, sentence structures, etc.–during online language processing, and that these expectations are informed by and reflect the statistics of previous linguistic experience. By generating expectations that reflect the distribution of actual events in the environment, comprehenders should, in principle, be able to reduce the average prediction error experienced during online processing, and thus process language efficiently (we return to this point in more detail shortly). But if the distribution of words or sentence structures varies according to individual speakers, dialects, genres, etc., then, at first blush, it is no longer clear that generating online linguistic expectations that reflect aggregate statistics over previous experience would be advantageous to the comprehender. In this paper, we sketch–in informal terms–a computational-level framework to guide research on this question. We argue that syntactic adaptation allows comprehenders’ *expectations* about the statistics of the environment to converge towards or even on the *actual* statistics, thus providing an explanation for why experience-based processing is advantageous despite the variability present in the statistics of the linguistic signal. We test this hypothesis for sentence comprehension (“parsing”), specifically reading, and find it confirmed: comprehenders are able to rapidly adapt to the statistics of novel linguistic environments. (For a formal account of phonetic adaptation that is closely related to the ideas developed here, see [14]; for the first generation of implementations and elaborations of the account outlined here, see [15]–[17].).

We build on and attempt to synthesize insights from three research traditions that have up until now proceeded largely in parallel: experience- or expectation-based theories of language comprehension [11], [18], [19], syntactic priming in comprehension [20]–[22], and psycholinguistic research exploring the link between processing and implicit learning [23]–[28]. We begin by briefly reviewing those aspects of these lines of work that are most relevant to our goals (which we return to in more detail in the general discussion).

The starting point for our investigation is the observation that comprehenders seem to take advantage of *statistical contingencies* in the input in order to process language [11]. For example, there is broad agreement that listeners are able to anticipate upcoming words and structures based on cues in the preceding parts of an utterance [29]–[34]. Statistical contingencies also play a crucial role in the processing of temporarily ambiguous material in so-called “garden path” sentences [11], [35]–[39]. For example, the past participle form of English verbs often gives rise to temporary ambiguities since these verbs may occur both as a main verb (MV), as in (1a), or as the verb in a relative clause (RC), as in (1c).

1. The experienced soldiers…
  - …warned about the dangers before the midnight raid.
  - …spoke about the dangers before the midnight raid.
  - …warned about the dangers conducted the midnight raid.
  - …who were told about the dangers conducted the midnight raid.

Sentences (1a) and (1c) are temporarily ambiguous (during *…warned about the dangers…*), but can be disambiguated toward the RC reading at *conducted* (1c). By contrast, (1b) is unambiguously an MV structure because *spoke* is unambiguously a past tense matrix verb. Similarly, (1d) is unambiguously an RC because of the relativizer *who*, which serves as an early disambiguating cue. Sentences like (1c) consistently elicit what are known as ambiguity or garden-path effects. That is, reading times (RTs) in the disambiguating region spike when the ambiguity is resolved towards the relative clause interpretation (1c), compared to unambiguous RCs (1d) [40]–[42]. No such ambiguity effect is found for ambiguous compared to unambiguous MVs. Experienced-based accounts predict the garden-path effect because verbs like *warned* are overwhelmingly more likely to occur with MVs than RCs in subjects’ previous experience, as evidenced in corpora of written and spoken language [9].

Findings like these constitute much of the empirical support for cue-based, competition, constraint satisfaction, and–in the most general terms–experience-based accounts of language processing [11], [12], [18], [19], [38], [43]–[49]. All of these accounts share the assumption that listeners’ previous language experience shapes the way they process language [13], [24]. That is, listeners are assumed to process language according to their *beliefs* or *expectations* about the language based on their previous experience. Beliefs or expectations can, in turn, be quantified as probability distributions computed over words, syntactic structures, etc. (Although many experience-based processing accounts are framed in terms of relative “activation”, rather than probabilistically, these two views can often be translated into one another [50]–[52]; see, for example, the discussion in [26]).

Many of these accounts share the often-implicit assumption that experience-based language processing is *efficient* [13], [19], [44], [45], [53]–[56]. Indeed, by anticipating linguistic events in proportion to their probability, we are able to minimize, on average, how surprised we are during language comprehension [19], [44], [56]. However, expectation-based processing will only be efficient to the extent that comprehenders’ expectations are sufficiently closely aligned with their interlocutors’ production preferences. This assumption is only warranted if at least one of the following two scenarios holds: (1) syntactic preferences are sufficiently stable between speakers, or (2) listeners can (rapidly) adapt to differences between speakers.

As discussed above, (1) is unlikely to be the case. Syntactic preferences vary significantly across linguistic environments. Thus, a comprehension system that places a premium on efficiently processing linguistic input will benefit from allowing expectations for linguistic events (words, structures, etc.) to be sensitive to speakers, genre, and other indexical factors. Because it is impossible that a comprehender will encounter an example of every possible environment before some relatively early point in life, and then use these experiences to guide subsequent comprehension, one way in which comprehenders might cope with variability in the input is to adapt their expectations to new linguistic environments. In other words, comprehenders may respond to variability in the environment by *learning* and *representing* that variability. This leaves us with the possibility of syntactic adaptation (i.e., scenario 2).

### Previous Work on Syntactic Adaptation

There is evidence that comprehenders, when faced with a novel linguistic environment, can continuously integrate expectations based on their previous linguistic experience with information they receive about the current linguistic environment. Preliminary evidence for this comes from work on syntactic priming, which shows that recent experience with syntactic structures can give rise to changes in how easily those structures are comprehended. This work has almost exclusively focused on the effect that a prime has on the comprehension of a target structure *immediately following* the prime [20], [22], [57]. This work thus leaves open whether there are *cumulative* effects of recent experience on language comprehension – a prerequisite for successful adaptation to the statistics of the current linguistic environment.

A small number of recent studies speaks to this issue [17], [58]–[60]. One particularly important piece of evidence comes from recent findings that, with sufficient exposure, listeners have access to syntactic expectations conditioned on specific talkers [58]. Similarly, there is evidence that repeated exposure over multiple days to certain syntactic structures improves readers’ ability to process them [28]. These studies suggest that cumulative recent experience affects how we process language, but leave several key questions open about exactly how comprehenders respond to linguistic environments with unexpected statistical properties, and exactly how insights from previous research–namely research on experience-based processing, syntactic priming, and statistical learning–can be synthesized to understand this process. In the section that follows, we outline our proposal before presenting two self-paced reading experiments that test the proposal.

### The Current Proposal

The primary goal of this paper is to investigate whether cumulative adaptation allows comprehenders to *converge towards*, or even *on*, the statistics of the current linguistic environment. If adaptation facilitates efficient processing, as we hypothesize, convergence is predicted (see also [26]). Convergence towards the statistics of the input would provide an account for the observation that talker-specific expectations affect sentence processing [58].

We propose that comprehenders enter a given linguistic environment with beliefs about the probabilities of syntactic structures. We assume a relatively atheoretical definition of “syntactic structure”; here, we rely on sentence types like those assumed by Roland et al. (2007) [9]. The proposals here are compatible with the representational assumptions underlying previous work in computational psycholinguistics, including connectionist models of syntactic production [23] and comprehension [24], as well as exemplar-based models of language use [61]. We assume that comprehenders’ beliefs about the distribution of syntactic structures can be quantified in terms of probabilistic expectations, or beliefs about a probability distribution. We define linguistic environments as any cue or combination of cues perceived by the comprehender to be related to the way linguistic events are distributed when those cues are present. Throughout this paper we assume that each behavioral experiment constitutes a novel linguistic environment from the perspective of subjects participating in that experiment. Analogously, we can consider individual talkers to constitute linguistic environments, since talker identity can provide information about which syntactic structures, for example, one is likely to encounter. It is in this sense that our proposal can capture talker-specific priming effects [58].

Coarse estimates of the parameters of the distributions comprising comprehenders’ beliefs prior to entering an environment can be obtained from corpora or norming studies. We propose that, upon entering a novel environment, comprehenders observe linguistic events that, by hypothesis, lead to changes in their beliefs about these distributions, causing convergence *towards* or, eventually, even *on* those of the objective distributions. That is, at least in cases in which a change in environment is recognized (e.g., because a new speaker is encountered), we predict that comprehenders are implicitly learning some approximation of the objective statistical structure of the novel linguistic environment. This adaptation allows comprehenders to reduce the processing costs that would otherwise result from un-adapted (and hence mistaken) syntactic expectations.

**Figure 1:**
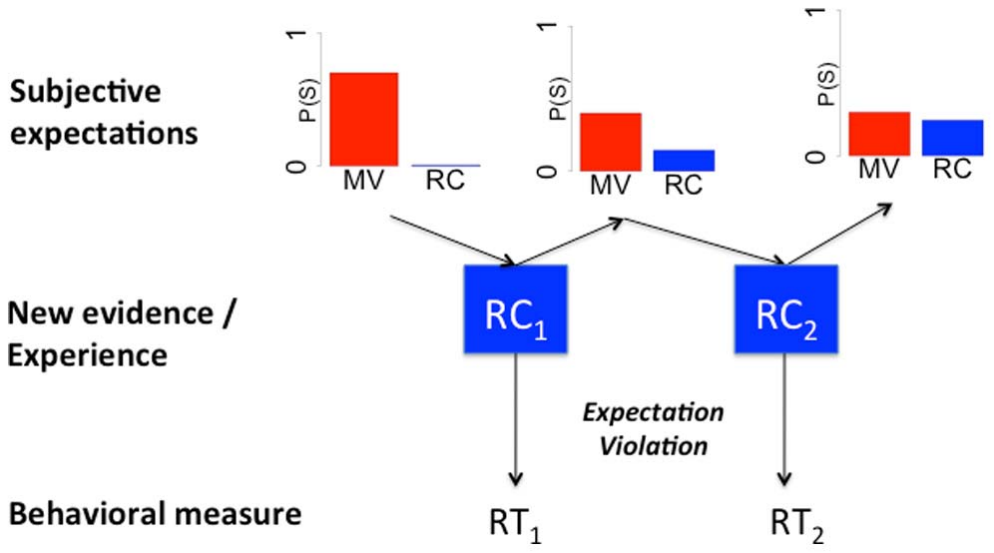
The current proposal. Schematization of the current proposal, illustrating the relationship between comprehenders’ beliefs, the effect that observations in a new linguistic environment have on those beliefs, and the manner in which behavioral measures such as reading times provide a window onto these changes.

Figure 1 illustrates this for an experiment in which a comprehender observes RC structures like in (1) above. Comprehenders enter the experimental environment and, based on their prior experience, have beliefs about the relative probabilities of MVs and RCs. For example, RC continuations are rather infrequent after past tense/participle ambiguous verb forms like those in (1c) above, whereas MV continuations are highly expected. For example, averaging across all ambiguous verbs considered in a recent large-scale corpus [9], the probability of an RC after an ambiguous verb form is smaller than.01, whereas that of an MV is larger than.6. This is illustrated by the subjective probability distribution in the top left of Figure 1 (the two bars do not sum to 1 since other types of continuation are possible). After observing an RC structure, comprehenders’ beliefs come to reflect this observation: the second distribution in the top row of Figure 1 assigns a slightly higher probability to RCs than the first.

Although we cannot directly observe comprehenders’ beliefs, we can measure RTs during the disambiguating region of RCs to obtain a behavioral measure reflecting comprehenders’ expectations about the relative probabilities of MVs and RCs: RTs during the disambiguating region in temporarily ambiguous relative to unambiguous RCs reflect processing difficulty [41], [62]. And, most relevant to our goals, this processing difficulty has been linked to the degree of expectation violation experienced when the initially preferred parse is no longer compatible with the observed word sequences [19], [44], [56]. By measuring changes in ambiguity effects as subjects’ experience with the experimental environment accumulates, we can obtain a behavioral index of the underlying adaptation process (changes in subjects’ beliefs, pictured in the top row of the figure). With this in mind we can now spell out the two predictions of the proposed framework that we seek to test (further predictions are derived in the general discussion).

Figure 1 illustrates the first key prediction: as experience with an *a priori* unexpected structure accumulates for a novel environment, RTs should reflect this experience, and the ambiguity effect observed for such sentences should decrease. This prediction is tested in Experiment 1.

The second key prediction is that, as experience with one structure accumulates and comprehenders thereby assign a higher probability to that structure, other structures competing with that structure for probability mass should come to be assigned a lower probability, and should therefore take longer to process. Specifically, if comprehenders experience an environment in which examples like (1) are always or nearly always disambiguated towards an RC continuation (i.e., the *a priori* unexpected structure), eventually, the *a priori* more expected structure (MVs) should become unexpected. With sufficient exposure, it should thus even be possible to elicit garden path effects on sentences that in everyday experience are highly frequent and expected. This prediction is tested in Experiment 2.

An additional goal of this article is to explore the time course over which syntactic adaptation unfolds. Specifically, as comprehenders receive more evidence about the distributional properties of a novel environment, how quickly do they adjust their expectations? For adaptation to best facilitate online language processing, we would expect relatively rapid effects of exposure on processing. Currently, little is known about how syntactic adaptation unfolds over the course of experience (but see [17], [58], [59]).

We can focus on specific portions of Figure 1 to see the link (and the differences) between the current proposal and some of the previous work guiding our proposal. First, if we focus only on the correlation between the prior probability distribution (estimated based on corpus or norming statistics) and RTs during the disambiguating region of RC structures, while ignoring how this probability distribution might change as a result of observations in the experiment, this corresponds to most previous work on expectation-based processing [11], [39]. Similarly, focusing on RTs on adjacent trials (RT_1_, RT_2_, etc.) in which similar structures were observed, while largely ignoring whatever underlying beliefs about the environment preceded these observations (as well as the fact that these observations in turn alter these underlying beliefs), would correspond to most previous work on syntactic priming in comprehension [20]–[22]. Finally, previous work on statistical learning [63]–[65] would correspond, in Figure 1, to probing (typically offline) whether subjects have learned the distribution of linguistic items in the experiment (e.g., what distribution the events in the middle layer–RC_1_, RC_2_–follow in the experiment). This work typically ignores whatever beliefs subjects might have had about the distribution of those elements *prior to* the experiment (though statistical learning experiments often employ linguistic materials, namely artificial languages, that at least implicitly allow the researcher to “control” the prior beliefs subjects have about those materials), as well as how this learning unfolded incrementally and cumulatively.

In the remainder of this paper, we present two self-paced reading experiments that test the predictions that follow from the account summarized in Figure 1. We describe the detailed predictions for each experiment below. The paper concludes by discussing the consequences of our experiments for current debates concerning the mechanism that mediates syntactic adaptation (implicit learning vs. short term activation), by offering some tentative comments on the possibility that adaptation of the kind observed here reflects a highly domain-general cognitive principle, and by briefly discussing potential methodological implications of our findings.

Before proceeding, a brief terminological note is in order: We use the terms “adapt” and “adaptation” to refer to the processes that enable comprehenders to adjust their linguistic expectations–conferred via previous experience–to a specific speaker or environment. In this context, it is important to note that the same or similar concepts have been discussed under a variety of different labels, often without further definition, including “entrainment” [66]–[68], “accommodation” [69]–[71], “alignment” [72], and, perhaps most importantly, “syntactic priming” or “structural priming”, at least where these terms have been used in the context of language comprehension [20]–[22], [73].

## Experiment 1

Experiments 1 and 2 use sentences like those illustrated in (1a)–(1d), repeated below as (2a)–(2d).

1. The experienced soldiers/…
  - …warned about the dangers/before the midnight/raid.
  - …spoke about the dangers/before the midnight/raid.
  - …warned about the dangers/conducted the midnight/raid.
  - …who were told about the dangers/conducted the midnight/raid.

Experiment 1 is essentially a replication of MacDonald et al. (1992) [41], with additional critical items added. Crucial to the questions under consideration here, the statistics of Experiment 1 differ strongly from the statistics of subjects’ prior experience with the language: on 50% of the trials containing a syntactic ambiguity, the ambiguity is resolved in accordance with the RC interpretation, thus strongly violating subjects’ prior expectations, as estimated from sentence norms or corpus statistics (for the particular verbs in our experiment, p(RC) = .008 and p(MV) = .7 according to Roland et al. (2007) [9]).

A detailed comparison of the statistics of the experiment and the statistics of subjects’ previous experience leads to two specific predictions. First, RCs are far more probable in Experiment 1 than in subjects’ previous experience (.5 as compared to.008, conditioning just on the verbs used in this experiment). If comprehenders adapt to this change in syntactic statistics, subjects should come to have increasingly stronger expectations for RCs, and this should be reflected in increasingly smaller ambiguity effects. The predicted change in reading times at the disambiguating region is further exaggerated due to the log-linear relation between linguistic probabilities and reading times [56]. That is, reading times increase linearly with surprisal (surprisal(x) = log (1/p(x)), [44]). Surprisal has also been found to be a good predictor of the amount of expectation shift experienced after a syntactic prime [25], [26]. That is, both processing data and learning data suggest that surprisal is a good approximation of the error signal experienced by comprehenders when processing linguistic information. While we assume a surprisal link function, any monotonic function relating linguistic probabilities to reading times correctly predicts the *ordering* of effects in Experiment 1 and 2.

**Figure 2:**
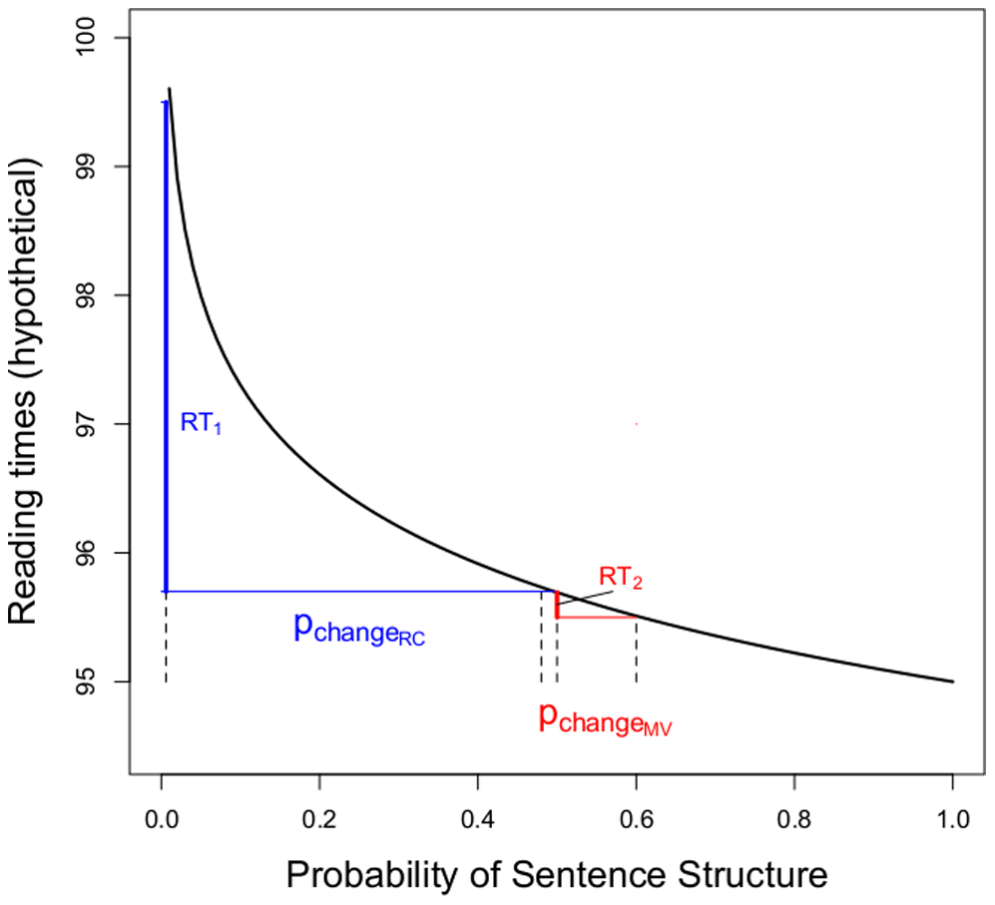
Relationship between reading times and linguistic probabilities. Illustration of the log-linear relationship between reading times and linguistic probabilities, following Smith and Levy (2013). We indicate the changes in probabilities (horizontal lines) for relative clauses (RCs; blue) and main verbs (MVs; red) for Experiment 1 and indicate the predicted changes in reading times for both (vertical lines).

The surprisal link function relating RTs to linguistic probabilities is illustrated in Figure 2. The blue segments in Figure 2 illustrate changes in hypothetical RTs for RC structures, from the *a priori* probabilities of these structures (.008 = 6.97 bits of surprisal) towards the probability of RCs in Experiment 1 (.5 = 1 bit of surprisal).

Second, in contrast to RCs, comprehenders’ subjective expectations for MVs should *de*crease. However, this change is predicted to be hardly detectable since the change in surprisal for MVs is predicted to be minimal (from.51 bits at the beginning of the experiment, when p(MV) = .7, to a maximum of 1 bit at the end; i.e. the predicted maximum increase in surprisal for MVs is less than 9% of the predicted maximum decrease in surprisal for RCs). This is illustrated by the red segments in Figure 2.

**Figure 3:**
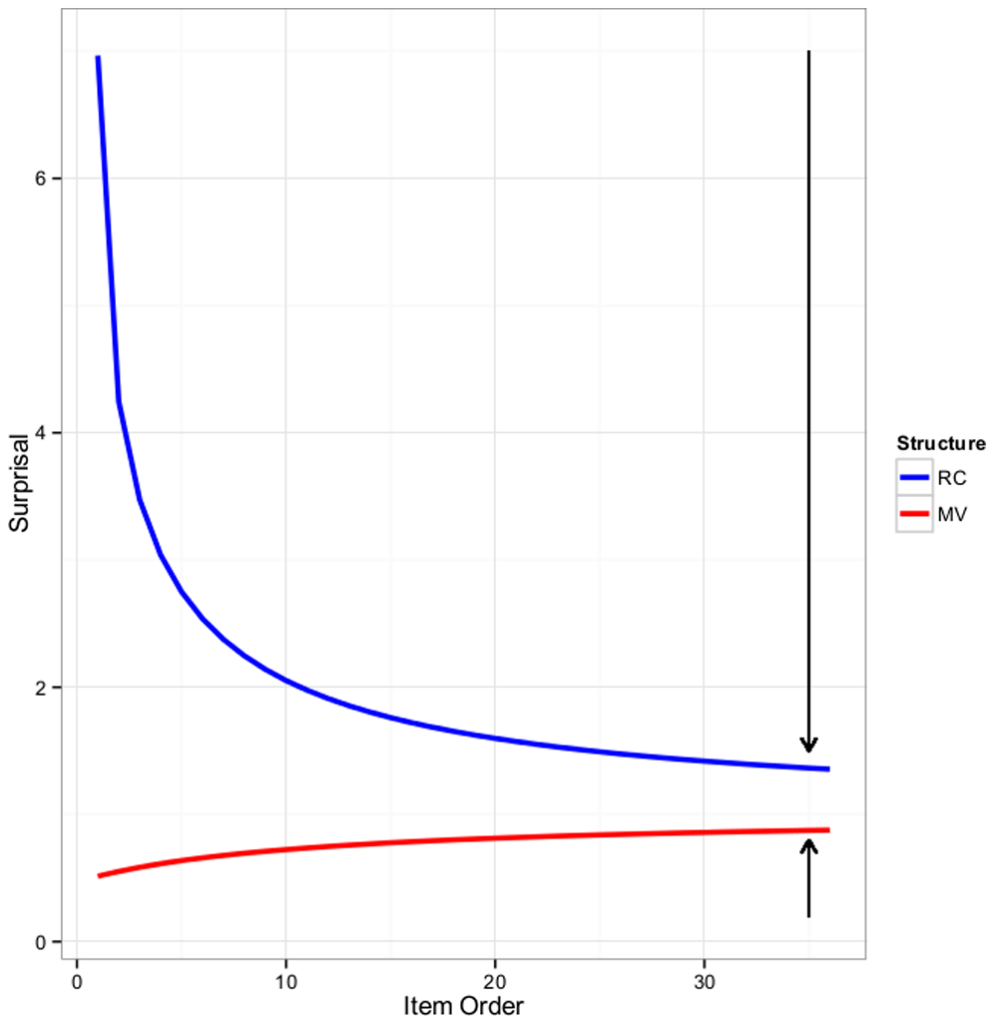
Predicted changes in surprisal for Experiment 1. Qualitative illustration of the changes in surprisal for MVs and RCs predicted by our account throughout Experiment 1. Black arrows indicate the predicted changes in surprisal at the end of Experiment 1: large changes for RCs (blue), but tiny changes for MVs (red).

Finally, Figure 3 illustrates the predicted change in surprisal (and hence the ambiguity effect) for both RCs and MVs throughout Experiment 1. The predictions in Figure 3 are derived from a Bayesian belief-updating model. Specifically, a beta-binomial belief-updating model was parameterized based on the relative frequencies of RCs and MVs in corpus counts (Roland et al., 2007 [9]) and one free parameter scaling these relative frequencies to pseudocounts. In the current context, these pseudocounts reflect the comprehenders’ beliefs about the extent to which prior experience with other linguistic environments will generalize to the current linguistic enviroment. We thus chose a very weak prior (consistent also with findings in Fine et al., 2010 [17]). Here we do not intend to model the data presented in our experiments. Figure 3 merely serves to illustrate the predictions of the framework we introduce in this paper. Similarly, the specific adaptated probability and surprisal values given in the text are meant to illustrate (rather than fit) quantitative differences.

### Method

#### Subjects

Seventy-three native English speakers participated in this study as part of a subject pool associated with an introductory psychology course. Subjects had normal or corrected to normal vision and were native speakers of English. One subject’s data were excluded due to errors in data recording.

#### Ethics statement

This study was conducted with the approval of the James Madison University research subjects review board. All subjects gave written consent and were compensated with extra credit in a psychology course in accordance with the policies of the James Madison University research subjects review board.

#### Materials

The materials in Experiment 1 were a modified version of those used by MacDonald et al. (1992) [41]. In their experiment, 24 items were created from triplets of verbs. For instance, the verb triplet *warned*, *spoke*, and *who were told* would correspond to an item with four versions, one version for each of the four conditions, as in (2).

In MacDonald et al. (1992) [41], eight MV/RC-ambiguous verbs such as *warned* were chosen to create 8 such triplets, and three items were derived from each triplet by varying the lexical content of the sentences. Because we are interested in an effect that, by hypothesis, unfolds over time, we introduced 4 more triplets that were created by Kemtes and Kemper (1997) [74] in order to extend the original MacDonald et al. sentence set, and constructed 3 items from each triplet. This added 12 items to the 24 from MacDonald et al. to yield a total of 36 items. In addition to the 36 critical items, subjects read 50 fillers.

Since, by hypothesis, comprehenders are sensitive to the distribution of syntactic structures, it was important to carefully choose the structures employed in the filler stimuli. One potential issue to address is that syntactic adaptation might be verb-specific, so that comprehenders adjust their expectations for a structure separately for each verb. Reason to believe that this might be the case comes from experiments that have found syntactic priming in comprehension only when the prime and target shared a verb [22]. To reduce interference from the syntactic statistics of fillers, fillers never contained any of the verbs used in critical items.

However, verb-independent priming has also been observed [20], [21], [75], suggesting that comprehenders can generalize syntactic adaptation across lexical contexts. Indeed, the design of Experiment 1 assumes such adaptation across lexical contexts since we assess adaptation effects across critical stimuli with different verbs. So, to minimize potential interference from filler stimuli, fillers never contained the RC or MV ambiguity or verb forms that (in their syntactic context) were ambiguous between a past tense and past participle interpretation. Specifically, most of the fillers used non-transitive verbs with sentence complements (e.g., *The newlywed husband hinted that he would like to go to the cabin for the weekend*), unambiguous passive verb forms (*The word processing program was written for novice users*), and infinitival verb forms (*The dogsitter decided to feed the dog the leftover dinner*).

Note that our qualitative predictions do not depend on the specific assumption that P_Exp1_(RC | …) = P_Exp1_(MV | …) = .5, so that our predictions should hold even if there is some moderate interference from the syntactic statistics of the filler stimuli. The prediction that we should see a significant reduction of the ambiguity effect for RCs, but no significant increase in the ambiguity effect for MVs, holds as long as –log P_Exp1_(RC)<< −log P_prior_(RC | …) and –log P_Exp1_(MV)≈−log P_prior_(MV | …). Given the statistics of Experiment 1 and the statistics corpus counts (Roland et al. (2007) [9]), this condition is very likely to hold.

#### Procedure

Subjects read sentences in a self-paced moving window display [76]. At the beginning of each trial, the sentence appeared on the screen with all non-space characters replaced by a dash. Subjects pressed the space bar using their dominant hand to view each consecutive word in the sentence. Durations between space bar presses were recorded. At each press of the space bar, the currently viewed word reverted to dashes as the next word was converted to letters. A yes/no comprehension question followed all experimental and filler sentences, with the correct answer to half of all comprehension questions being “yes”.

### Results

#### Data coding and exclusions

All raw RTs that were abnormally low (below 100 ms) or abnormally high (above 2000 ms) were removed, resulting in a less than 1% data loss. Length-corrected RTs [77] were computed by regressing the remaining raw RTs onto word length using linear mixed effects regression. In addition to a single main effect of word length, this model included a random intercept for subject, and a by-subject random slope for length (these random effects allow the model to discount mean differences in raw RT across subjects as well as variable sensitivity to the effect of word length across subjects). The residuals of this model, length-corrected RTs, served as the dependent variable in all analyses reported below. Qualitatively identical results are obtained if raw RTs are used instead.

#### Analysis

**Figure 4:**
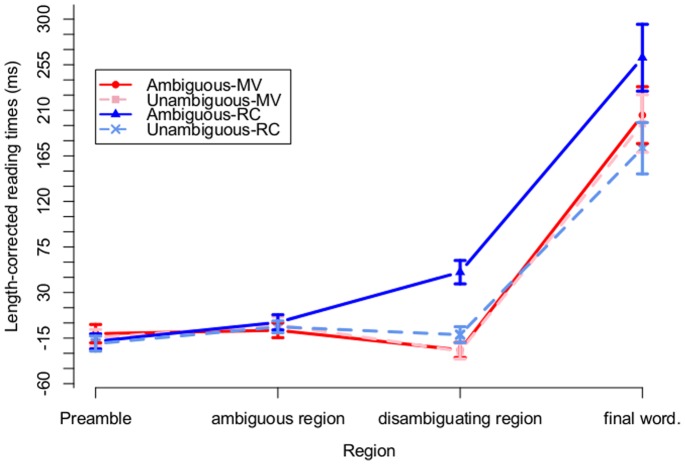
By-region reading times for Experiment 1. Mean length-corrected reading times at each sentence region (indicated in (2)) for all conditions in Experiment 1. Error bars represent 95% confidence intervals on the mean.

Although the sentences were read one word at a time, for the purposes of analysis, we segmented sentences into regions indicated by the forward slashes in (2) above. These regions are the same used in the analyses reported by MacDonald et al. (1992) [41]. We begin by plotting by-region mean length-corrected RTs in Figure 4. This figure serves to demonstrate that, before looking for evidence of syntactic adaptation, we have replicated the garden path effects found in previous work. Consistent with previous research, Figure 4 shows larger RTs for ambiguous (dark, solid lines) relative to unambiguous (light, dashed lines) sentences, and this difference is driven completely by the RC sentences (blue lines). This was also confirmed by our analysis, which we report next.

Length-corrected RTs at the disambiguating region (underlined in (2)) were regressed onto the full factorial design (i.e., all main effects and interactions) of (a) sentence type (RC vs. MV), (b) ambiguity (ambiguous vs. unambiguous), and (c) item order (from 1–36). In order to control for task adaptation (i.e., an overall speed-up in RTs across all regions due to increasing familiarity with the self-paced reading paradigm), we included (d) a main effect of log-transformed stimulus order. Stimulus order differs from item order in that it is an index of when the item was presented relative to both items and fillers. Stimulus order therefore captures essentially how long subjects have been doing the experiment, whereas item order captures the distribution over MVs and RCs observed by subjects at a given point in the experiment. The results reported below hold with or without this predictor in the analysis and regardless of whether it is log-transformed. Finally, the model included the maximal random effects structure justified by the data based on model comparison. All results reported below also hold if the model includes the fullest random effects structure that still allows the model to converge (see Appendix S1 for a detailed description of the modeling procedure).

The structure of the regression model thus includes (1) a predictor capturing *a priori* expectations about the linguistic environment–in this case, a two-way interaction between sentence type and ambiguity–and (2) a term capturing the way these expectations change over the course of the experiment–the three-way interaction between sentence type, ambiguity, and item order. All predictors were centered in order to reduce multicollinearity between higher order interactions. Multicollinearity remained low (*r*<.2), with the exception of unsurprisingly high multicollinearity between the coefficients for item order and log-transformed stimulus order (*r = *.8). Because multicollinearity only affects the standard error estimates of predictors that are collinear, and because these two main effects themselves are not of central theoretical interest, multicollinearity in this case does not compromise the results reported below.

The results of this model are summarized in Table 1. Replicating previous studies, we found a significant sentence type by ambiguity interaction–reading times were higher in ambiguous than in unambiguous sentences, but this difference was driven by the RC sentences (**β** = −13.7; *p*<.05). As expected, there was a significant main effect of log stimulus order (**β** = −84.4; *p*<.05).

**Table 1:** Summary of model results at each sentence region in Experiment 1.

Predictor		Preamble		Ambiguousregion		Disambiguatingregion		Finalword
**Log stimulus order**	**−54**	**(−2.6)**	**−83**	**(−7.2)**	**−84**	**(−5.8)**	**−196**	**(−5)**
**Ambiguity**	−1	(−.6)	.6	(.2)	**−15**	**(−4.7)**	**−28**	**(−3.4)**
**Sentence type**	**−5**	**(−2.5)**	−.2	(−.1)	**21**	**(6)**	9	(.8)
**Item order**	−1	(−1)	−.2	(−.3)	.3	(.4)	−2.1	(−.9)
**Sentence type : Ambiguity**	1	(.7)	−.9	(−.4)	**−14**	**(−6.4)**	**−24**	**(−3)**
**Sentence type : Item order**	−.1	(−.5)	−3	(−1.1)	**−1**	**(−3.5)**	−.6	(−.6)
**Ambiguity : Item order**	.1	(.6)	.2	(1.1)	**.6**	**(2.7)**	.6	(.8)
**Sent. Type : Ambiguity : Item order**	.2	(1)	.2	(1)	**.54**	**(2.2)**	**1.5**	**(2)**

Summary of coefficients (and t-values) at each sentence region for each of the models reported in Experiment 1. For models fit to data sets of this size, coefficients with t-values having an absolute value of 1.96 or greater are significant at *p*≤.05. Significant effects are in bold.

The crucial predictor in this experiment is the three-way interaction between sentence type, ambiguity, and item order. This predictor captures how the two-way interaction between sentence type and ambiguity changes as subjects are exposed to the statistics of Experiment 1. If subjects are adapting to environment-specific statistics, they should assign increasingly higher probability to the RC structure over the course of the experiment. This should lead to increasingly smaller ambiguity effects for RCs (i.e., the difference in length-corrected reading times, at the point of disambiguation, between ambiguous and unambiguous RCs should decrease). Concretely, because the two-way interaction capturing the processing cost of the RC structure has a coefficient with a negative sign, adaptation would surface as a three-way interaction with a positive coefficient. A positive coefficient on the three-way interaction indicates that the strength of the two-way interaction is decreasing in magnitude as the experiment progresses. This is what we observed (**β** *= *.54; *p*<.05).

**Figure 5:**
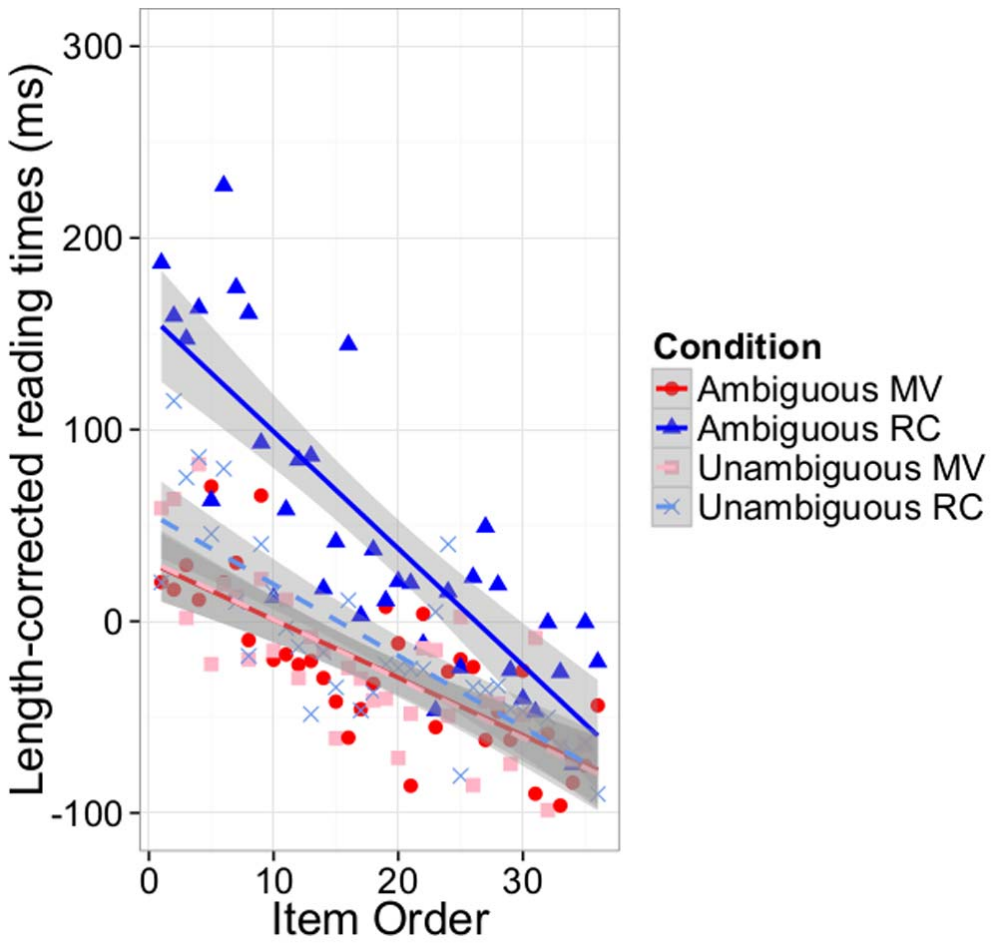
Adaptation effect for Experiment 1. Change in length-corrected RTs plotted against item order, for ambiguous (dark lines) and unambiguous (light lines) MVs (red) and RCs (blue). Gray shaded regions correspond to 95% confidence intervals on the slopes.

Figure 5 illustrates the three-way interaction by plotting how RTs during the disambiguating region change over the course of the experiment for ambiguous and unambiguous RCs and MVs. Figure 5 reveals the predicted asymmetry in the adaptation effect: the ambiguity effect for RCs decreases sharply over the course of the experiment; this change is so drastic that, by the end of the experiment, the ambiguity effect for RCs is not statistically distinguishable from that for MVs. By contrast, the ambiguity effect for MVs changes little over the course of the experiment, increasing slightly but not significantly. This was confirmed by simple effect analyses–the interaction between ambiguity and item order is significant for RCs (p<.05) but not for MVs (p = .6).

Overlap in the ambiguity-producing verb was not systematically controlled in this preliminary experiment, such that a small number of verbs appeared 2 times in the materials (this did not hold for the materials used by [41]. Because a number of previous studies have suggested that syntactic priming in comprehension depends on prime and target sentences sharing the same verb [22]; but see [20], [21]), this raises the question whether the cumulative priming effects observed in this experiment were due to verb repetition. To address this possibility, we performed the same analysis reported above after removing items whose verb had already been seen by that subject. This analysis yielded qualitatively identical results to those reported above, including significant main effects of log stimulus order, ambiguity, sentence type, two-way interactions between ambiguity and sentence type and sentence type and item order. The ambiguity by item order interaction was marginally significant (p = .09). Finally, the crucial three-way interaction between ambiguity, sentence type, and item order was nearly significant (p = .058). The reduction in the reliability of the effect is very likely due to the loss of power (caused by about 20% less data for this analysis).

### Discussion

The central finding from Experiment 1 is shown in Figure 5, which demonstrates the manner in which linguistic experience rapidly, incrementally, and cumulatively shapes subjects’ expectations for multiple syntactic structures. In the most general terms, Experiment 1 demonstrates that readers incrementally integrate recent processing experience (reflecting the statistics of the experiment) with their previous language experience. We interpret this as the result of subjects’ ability to rapidly adjust their syntactic expectations to approximate the statistics of the immediate linguistic environment.

We made an effort in the analysis of Experiment 1 to rule out the possibility that the adaptation effects observed here are due to “task adaptation”, i.e., the possibility that subjects are adapting to the self-paced reading task itself, causing RTs to reach a floor value. We showed (a) that the adaptation effect reported here holds even after controlling for main effects of item order and log-transformed stimulus order, and (b) that the predicted effect only surfaces at the predicted region (i.e., during the disambiguating region). Experiment 2 directly compares the effect of experience with the task itself and experience with the relevant structures and provides further evidence that the effect reported for Experiment 1 is not due to task adaptation.

That adaptation seems to be affecting the comprehension of both structures is relevant since our characterization of adaptation as the continuous, rapid adjustment of syntactic expectations leads to the specific prediction that if a comprehender’s expectations for two syntactic structures are not independent (i.e., if the probabilities that a comprehender assigns to those structures are not independent, as in the case of MVs and RCs), then changes in expectations for one structure should lead to changes in expectations for the other. The results from Experiment 1 are consistent with this prediction, since the slope characterizing the change in the ambiguity effect over the course of the experiment is negative for RCs and positive for MVs. It is not surprising that the effect for MVs did not reach significance since, as we mentioned above, the statistics of Experiment 1 implied that a large shift in expectations was expected for RCs, but a small shift for MVs.

Experiment 2 builds on Experiment 1 and employs a design intended to lead to stronger adaptation effects by creating larger shifts in the local statistics of the linguistic environment, and therefore to increase the probability of observing adaptation effects for MVs as well as RCs.

## Experiment 2

In Experiment 2, we again take advantage of the temporary ambiguity between MVs and RCs as a window onto subjects’ changing syntactic expectations. The primary motivation for Experiment 2 is to address a critical question that remains open after Experiment 1. Namely, in the absence of a reliable change in the ambiguity effect for MVs, the results are compatible with an explanation under which subjects are adapting to the experiment, but where this adaptation does not involve converging towards the statistics of the input.

For example, it is possible that each exposure to a syntactic structure leads to a constant increase in activation of that structure, which in turn facilitates processing of that structure, lowering RTs. This explanation would predict that the ambiguity effect for both RCs and MVs should decrease, whereas only the former was observed. However, the absence of a reduction of the MV ambiguity effect might also simply be due to the fact that there was no ambiguity effect for MVs to begin with. In this view, recent exposure to a structure can only lead to faster processing of that structure. If, on the other hand, comprehenders indeed adapt their expectations so as to reflect the statistics of the current environment, we would expect that it should, in principle, be possible to decrease the relative expectation for a structure enough to cause *slower* processing of that structure (after taking into account additional structure-insensitive task adaptation).

Experiment 2 employs a between-subject block design to address this question. Subjects were assigned to one of two groups, which we will call the Filler-First and the RC-First groups. In both groups, subjects were exposed to three blocks of sentences. The composition of the materials in each block, for each group, is shown in Table 2.

**Table 2:** Summary of the design and materials used in Experiment 2.

Group	Block 1	Block 2	Block 3
**RC-First (n = 40)**	16 RCs (8 ambiguous)	10 RCs (5 ambiguous) +20 fillers	10 MVs (5 ambiguous) +15 fillers
**Filler-First (n = 40)**	16 fillers	10 RCs (5 ambiguous) +20 fillers	10 MVs (5 ambiguous) +15 fillers

Cells summarize the properties of the sentences read by each group in each block in Experiment 2.

**Figure 6:**
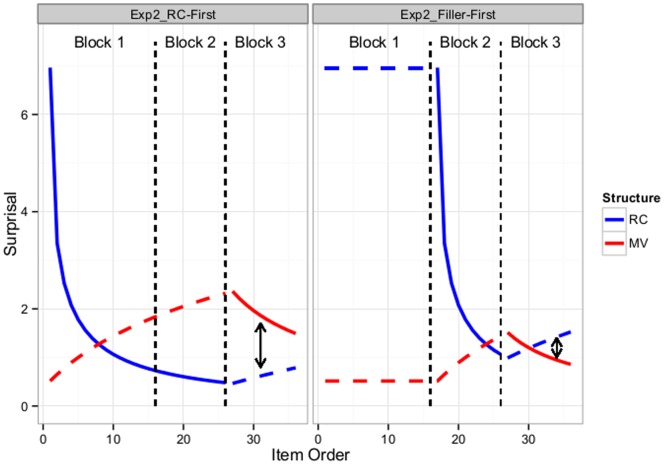
Predicted changes in surprisal for Experiment 2. Predicted changes in surprisal for the RC-First (left panel) and Filler-First (right panel) groups for both RCs (blue) and MVs (red). Dashed lines indicate predicted changes in surprisal of structures not present in a given block (e.g., the surprisal of MVs is predicted to change throughout Blocks 1 and 2 as a result of the occurrence of RCs, but no MVs actually occur in those blocks). Black arrows indicate the average predicted surprisal of MVs relative to RCs during Block 3.

Subjects in the RC-First group read 16 RCs in Block 1, 10 RCs in Block 2, and, finally, 10 MVs in Block 3. Subjects in the Filler-First group received identical experience, except these subjects saw only fillers (i.e., sentences in which the MV/RC ambiguity never arises) instead of RCs in Block 1. Figure 6 illustrates the predicted change in surprisal (and hence the ambiguity effect) for both RCs and MVs throughout the three blocks of Experiment 2, using the same method as in Figure 3. Next, we spell out three predictions Experiment 2 seeks to test.

First and foremost, we now expect to see adaptation effects for MVs, to be measured in Block 3. The surprisal for MVs should be largest for the RC-First group: at the onset of Block 3, MVs should have a subjective probability that is close to 0. Following the same method we used to derive the predictions in Figure 3, the probability of an MV at the onset of Block 3 should be about.019 (3.99 bits of surprisal, up from.51 bits at the onset of Experiment 2). The *average* surprisal of MVs in Block 3 should be about 1.86 bits, compared to 1.12 bits for subjects in the Filler-First group. For comparison, the average surprisal of MVs during Experiment 1 was.76 bits (in all cases, up from.51 bits at the onset of Experiments 1 and 2).

Figure 6 also suggests that, for the RC-First group, MVs should elicit even more surprisal than RCs. In other words, if our framework is on the right track, we might find that subjects in the RC-First group are garden pathed by MVs, rather than RCs, reversing –for this linguistic environment–the typically-observed RC disadvantage.

The design of Experiment 2 also allows us to test two additional predictions. First, we predict that the ambiguity effect for RCs will be diminished with increasing exposure to RCs from Block 1 to Block 2 for the RC-First group. This would conceptually replicate Experiment 1. Moreover, we predict that the ambiguity effect for RCs during Block 2 for the Filler-First group will be greater than that of the RC-First group. If the effects observed in Experiment 1 are due to task adaptation or fatigue, then the ambiguity effect for RCs in Block 2 should be the same for both the Filler-First and the RC-First group. In other words, reading a given number of sentences should have the same effect on RTs regardless of the content of those sentences. On the other hand, if the ambiguity effect is reduced in Block 2 for the RC-First group, but there is still a significant ambiguity effect in Block 2 for the Filler-First group, then this would suggest that–perhaps in addition to something like task adaptation–subjects are indeed adapting their expectations depending on the statistics of the linguistic environment.

### Method

#### Subjects

80 subjects were recruited from the University of Rochester community.

#### Ethics statement

This study was conducted with the approval of the University of Rochester research subjects review board. All subjects gave written consent and received $10 for their participation according to the policies set forth by the University of Rochester research subjects review board.

#### Materials

Subjects read a total of 71 sentences over 3 blocks (as outlined in Table 2). RC and MV sentences were created that followed the same template as the critical items from Experiment 1. Two experimental lists were constructed for each group that counter-balanced the conditions (ambiguous vs. unambiguous) for the sentence type (MV or RC) used within each block (indicated in Table 2). For the RC-First group, items in Block 1 contained 16 unique verbs. Block 2 contained 10 verbs that were a subset of the 16 used in Block 1 (only the verbs, but not the remainder of the sentence overlapped). Ambiguous items in Block 3 contained the same verbs used in the ambiguous items in Block 2, while unambiguous items in Block 3 had not yet appeared in the experiment (since no MVs had been seen at all). Blocks 2 and 3 were identical across groups in all respects.

Following Experiment 1, filler sentences never contained verbs that could exhibit the ambiguity. (e.g., because they contained present tense verbs, auxiliaries, infinitives, or past tense verbs with only intransitive interpretations, as in *The chess match lasted for hours and finally ended in a stale mate.*). All stimuli from Experiment 2, including fillers, are provided in Appendix S2.

Finally, it is important to note that the block structure of the experiment was entirely implicit. From the perspective of the subjects, they simply read 71 sentences without breaks or any other explicit or implicit indications of the block structure of the experiment.

#### Procedure

The exact same procedure was used for Experiment 2 as in Experiment 1. Experimental blocks, as depicted in Table 2, were not overtly indicated to subjects.

### Results

#### Data coding and exclusions

As was the case for Experiment 1, we first removed all RTs below 100 ms or above 2000 ms, resulting in ∼1% data loss. Length-corrected RTs were obtained using the same procedure described for Experiment 1.

#### Analysis

We present three separate analyses, corresponding to separate questions the current study was intended to address.

##### Question 1

The central prediction of interest in our analysis is whether the ambiguity effect for MVs is greater for subjects who have read more RCs, i.e., for subjects in the RC-First group. In the strongest case, this would mean that repeated exposure to an *a priori* unexpected structure (RCs) eventually leads to an ambiguity effect for the *a priori* highly expected structure (MVs). We regressed length corrected RTs during the disambiguating region of sentences read during Block 3 onto ambiguity (ambiguous MV vs. unambiguous MV), group (RC-First vs. Filler-First), and the interaction between these variables. Again, we included the maximal random effects structure justified by the data. Multicollinearity remained low (<.2), except for mild multicollinearity between the ambiguity by group interaction and the main effect of group (r = .3).

**Figure 7:**
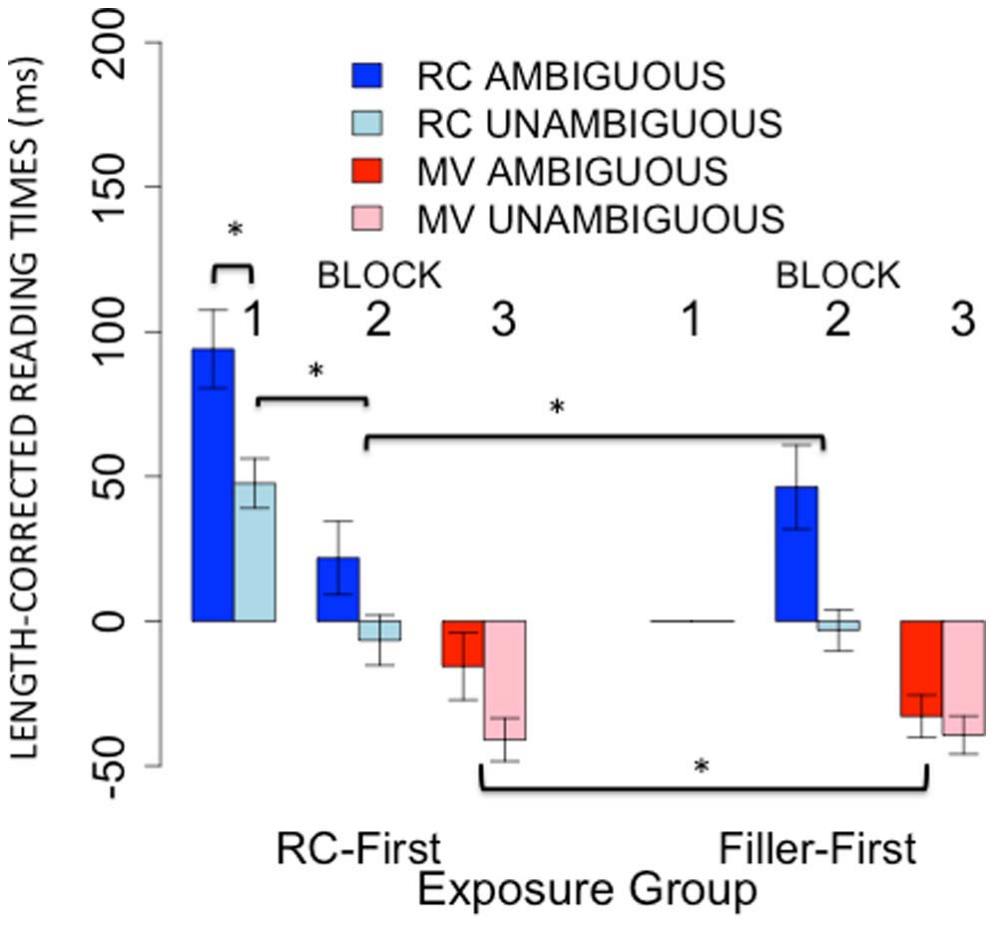
Results of Experiment 2. Mean length-corrected reading times in each block for the RC-First and Filler-First experimental groups. Error bars represent 95% confidence intervals on the means. Brackets with asterisks (*) indicate significant comparisons. For details, see main text.

There was a main effect of ambiguity, such that ambiguous MVs were read more slowly than unambiguous MVs (**β** = 8, p<.05). The main effect of group did not reach significance (**β** = 4, p = .3). Crucially, however, the two-way interaction between ambiguity and group was significant (**β** = 5, p<.05): the ambiguity effect for MVs during Block 3 was greater for the RC-First group than for the Filler-First group (because the interaction was collinear with the main effect, of group, we confirmed that the predictor was significant at p<.05 using model comparison based on log likelihood ratios). In other words, subjects who read more RCs subsequently experienced an increase in the ambiguity effect for MVs. Simple effect analysis confirmed that the ambiguity effect was significant for subjects in the RC-First group (**β** = 13, p<.001), but not subjects in the Filler-First group (**β** = 3, p = .3). This pattern is visualized in Figure 7 in the two pairs of red bars.

##### Question 2

Second, in order to provide a conceptual replication of Experiment 1, we asked whether the ambiguity effect in the RC-First group diminished from Block 1 to Block 2. We regressed length-corrected RTs during the disambiguating region during blocks 1 and 2 in the RC-First group onto ambiguity (ambiguous vs. unambiguous), Block (Block 1 vs. Block 2), and the two-way interaction between these predictors. The model also included the maximal random effects structure justified by the data. Multicollinearity remained low (<.02).

There was a significant effect of ambiguity (**β** = 20, p<.05): as in Experiment 1, ambiguous RCs were read more slowly than unambiguous RCs. There was also a significant main effect of block (**β** = −63, p<.05): subjects read faster during the second block relative to the first block. Finally, the interaction between these two variables, capturing the change in the ambiguity effect from Block 1 to Block 2, was in the predicted direction and trended towards but did not reach significance (**β** = −9, p = .2).

This lack of an effect was likely due to the reduced power of the type of analysis conducted for Experiment 2 (comparing aggregate reading times across two discrete blocks). “Binning” a continuous predictor into a binary contrast can increase the statistical power to detect an effect of that predictor when a) the relationship between the predictor and the dependent variable is monotonic, and b) the samples are drawn from the ends of the continuum. While a) is assumed to hold here, b) is clearly wrong: samples were drawn uniformly from the entire range of Item Order. To test this hypothesis, we took data from Blocks 1 and 2 for the RC-First group and submitted it to the same analysis reported for Experiment 1, regressing length-corrected RTs during the disambiguating region onto ambiguity (ambiguous vs. unambiguous) and item order (how many RCs have been seen at a given point in the experiment), and the interaction of these two variables. We also included a main effect of log stimulus order, as well as the maximum random effects structure justified by the data. We replicated the main effects of ambiguity (**β** = −39, p<.05) and log stimulus order (**β** = −176, p = .1). Crucially, the two-way interaction between ambiguity and item order was significant (**β** = 2, p<.05, after Bonferroni correction for multiple comparisons), replicating Experiment 1. In summary, subjects who read more RCs subsequently experienced both a reduction in the ambiguity effect for RCs and an increase in the ambiguity effect for MVs.

##### Question 3

Finally, Experiment 2 allows us to more directly address concerns that task adaptation or fatigue caused the effect for RCs observed in Experiment 1. To address this, we ask whether the ambiguity effect in Block 2 is greater for the RC-First group than for the Filler-First group. We regressed length-corrected RTs during the disambiguating region onto group (RC-First vs. Filler-First), ambiguity (ambiguous vs. unambiguous), and the interaction between these two variables. The model included the maximal random effects structure justified by the data, and multicollinearity remained low (<.3).

Again, there was a main effect of ambiguity, such that ambiguous RCs were read overall more slowly than unambiguous RCs (**β** = 19, p<.05). There was also a main effect of group: subjects in the RC-First group had overall faster reading times (**β** = −7, p<.05). Crucially, the two-way interaction between ambiguity and group was marginally significant (**β** = −5, p = .08): the ambiguity effect was smaller in the RC-First group than in the Filler-First group. This argues against an explanation in terms of fatigue since reading a block of filler sentences (i.e., sentences containing neither RCs nor verbs that create the MV/RC ambiguity) does not reduce the processing cost of RCs to the same extent that reading a block of RCs does. This result can be seen by examining the pairs of bars corresponding to block 2 for both groups in Figure 7.

The results of the analyses employed to address the three questions of interest in this experiment, when fit separately to each sentence region, are summarized in Table 3. Except for effects of block that surfaced in all regions for the second analysis, all effects surfaced in the disambiguating region or in the final word (since experimental effects in self-paced reading experiments can often “spill over” to regions neighboring those where the effect occurs).

**Table 3:** Summary of model results at each sentence region in Experiment 2.

Predictor		Preamble		Ambiguous region		Disambiguating region		Final word
**Question 1**								
**Ambiguity**	2	(.9)	2	(.8)	**8**	**(3)**	2	(.4)
**Group**	−1	(−.2)	3	(.7)	4	(1)	−6	(−.5)
**Ambiguity : Group**	.8	(.3)	−1	(−.4)	**5**	**(2)**	−1	(−.2)
**Question 2**								
**Ambiguity**	−5	(−1.5)	5	(1.8)	**20**	**(4)**	**26**	**(3)**
**Block**	**−57**	**(−5)**	**−53**	**(−5)**	**−63**	**(−4)**	**−114**	**(−5)**
**Ambiguity : Block**	7	(1)	−1	(−.1)	−9	(−1.3)	−9	(−.5)
**Question 3**								
**Ambiguity**	−2	(.8)	.6	(.2)	**19**	**(3)**	**40**	**(3)**
**Group**	−6	(−1.4)	−5	(−1.6)	**−7**	**(−2)**	**−40**	**(−3)**
**Ambiguity : Group**	2	(.8)	4	(1)	−5	(−1.7)	**−20**	**(−2)**

Summary of coefficients (and t-values) at each sentence region for each of the models reported in Experiment 2. For models fit to data sets of this size, coefficients with t-values having an absolute value of 1.96 or greater are significant at *p*≤.05. Significant effects are in bold.

## General Discussion

In this paper we tested the hypothesis that language comprehenders are able to adapt their syntactic expectations to novel linguistic environments according to the statistics of those environments. In two reading experiments, we provided subjects with experience with distributions of syntactic structures that diverged sharply from their previous experience with English. We predicted that subjects would adapt their expectations (as reflected in changes in RTs) according to their cumulative recent experience. As predicted, in Experiment 1 subjects came to process *a priori* infrequent structures that had initially produced longer RTs more quickly when those structures were frequent in the experiment. Experiment 2 replicated this effect and went a step further: there, subjects not only came to process an *a priori* infrequent structure more quickly, but also came to process an *a priori* frequent structure significantly more slowly when it was infrequent in the experiment, producing a garden-path effect for MVs. Our experiments suggest that readers are capable of adapting to the *relative* frequencies (or probabilities) of syntactic structures in the current linguistic environment. Specifically, the relative size of the ambiguity effects for MVs and RCs in Experiments 1 and 2 is expected if readers adapt their *a priori* beliefs to successfully converge towards the experiment-specific statistics. In the remainder of this paper, we discuss the implications of our findings for ongoing debates surrounding syntactic processing and syntactic priming, for the recently articulated notion that adaptation, writ large, constitutes one of the central principles of human perception and cognition, and for methodological questions in language processing experiments.

### Previous Evidence for Rapid Syntactic Adaptation

The experiments presented here build on work in syntactic priming [20]–[22]. We have asked specifically whether priming can be characterized as a form of expectation adaptation whereby each syntactic structure that a comprehender encounters counts as *a piece of evidence* that the comprehender uses to update their expectations about the distribution over syntactic structures. For that reason, we were interested in the cumulative effect of recent experience on syntactic expectations (e.g., as reflected in the processing difficulty experienced during temporarily ambiguous sentences) and, specifically, whether comprehenders’ expectations gradually converge towards the statistics of the linguistic environment as a consequence of cumulative experience. A number of previous findings speak to this issue, although they are not necessarily framed as such.

Kaschak and Glenberg (2004) [59] find that when comprehenders are exposed to structures they do not already know –in their study, the “needs+participle” construction, as in *this car needs washed*, which is grammatical only in some dialects of English–then the initial processing cost incurred in processing such structures seems to rapidly and cumulatively diminish (see also [78]). This observation is in line with the hypothesis advanced here, and suggests that the facilitation effect observed in Experiment 1 extends to other syntactic structures (although it is an open question whether the acquisition of novel structures like those used by Kaschak and Glenberg is due to the same mechanisms underlying expectation adaptation for syntactic structures that are already part of the comprehender’s grammar).

Also relevant is a recent study by Farmer and colleagues [60]. In their experiment, sentence frames were designed to produce a strong expectation for either a noun phrase continuation such as *the marble* (3a), or an infinitival complement continuation such as *to vary* (3b).

(3a) The curious young boy saved … (Noun-biased sentence frame)

(3b) The very old man attempted … (Verb-biased sentence frame)

The expectation for either continuation was produced by using matrix verbs (such as *saved* or *attempted*) that, in norming studies, were followed almost exclusively by either an NP in the noun case or an infinitival complement in the verb case. Of interest, Farmer et al. observed that when the noun- and verb-biased sentences were intermixed with one another, the strength of the bias for either an NP or verb, given the matrix verb, decreased over the course of the experiment. As a result of the strong structural overlap in the sentence frames across both the noun- and the verb-biased items, Farmer and colleagues argued that the sentence frames, including the bias-conferring matrix verb, were progressively less predictive of the grammatical category of the upcoming words in the sentences, and that subjects learned this regularity.

Recent work by Kamide offers further evidence for syntactic adaptation, and directly addresses a question left open by the other studies cited–namely, whether adaptation is environment-specific. Kamide (2012) [58] shows that comprehenders exhibit talker-specific syntactic priming. In her study, subjects heard sentences with temporary attachment ambiguities such as *The uncle of the girl who will ride…*, where either *uncle* or *girl* can be interpreted as the antecedent of the relative clause *who will ride*. Sentences were subsequently disambiguated on the basis of real-world knowledge when the sentence continued either with *…the carousel…* (which leads to interpreting *the girl* as the antecedent) or with *…the motorcycle…*. The display contained images of both a carousel and a motorcycle, so that subjects’ fixations during the ambiguous region could be interpreted as an index of which attachment the subject expected. The crucial manipulation in this study was that critical items were produced by two talkers with distinctive attachment “styles”: one talker always produced high attachments (*The uncle of the girl who will ride the motorcycle is from France*); the other always produced low attachments (*The uncle of the girl who will ride the carousel is from France*). Kamide presented her sentences in blocks and argued that, after exposure to the different talker styles, subjects learned the talkers’ production preferences, and made anticipatory saccades to the appropriate objects in the display depending on which talker they heard. One question left open by Kamide’s experiment is whether listeners integrated their previous expectations with the speaker-specific syntactic statistics they received during the exposure phase or whether they merely learned to associate (perhaps even categorically) each speaker’s voice with a specific structure. Only the former case would constitute the type of implicit statistical learning we have provided evidence for. Our results suggest that incremental expectation adaptation *might* underlie Kamide’s result, but future work is necessary to address this question directly.

The results reported in Farmer et al. (2011) [60], Kaschak and Glenberg (2004) [59], and Kamide (2012) [58] provide further support for the hypothesis that comprehenders can rapidly adapt to changes in the statistics of the environment. However, implicit learning and convergence on the statistics of the input are not the only explanations that could account for the results above. Indeed, Kaschak and Glenberg (2004) [59] offer an alternative explanation for their results. Specifically, under their episodic processing proposal, syntactic processing requires retrieving lexically specific representations of syntactic structures from memory, and this retrieval “episode” facilitates subsequent retrieval of the same representation (Kaschak & Glenberg, 2004, p. 452; building on work on skill acqusition, cf. [79], [80]). Importantly, this account makes predictions about the effect of exposure to structures that participate in an *ambiguity*, which the experiments in the current paper, as well as those reported in Kaschak and Glenberg, utilized.

Kaschak and Glenberg (2004) investigate how exposure to non-standard *needs+*participle sentences, as in (4a), which are common in some American dialects, affects processing of the standard use of *needs*+participle, as in (4b), where the participle is a modifier to the noun.

(4a) The meal needs cooked given that dinner is in an hour. (non-standard *needs*)

(4b) The meal needs cooked vegetables to make it complete. (modifier)

The episodic processing proposal advanced by Kaschak and Glenberg predicts that readers will initially mis-parse sentences such as (4a) as having the more frequent modifier structure (i.e., they are garden-pathed). This retrieval should subsequently *facilitate* processing of that modifier structure, even though the modifier interpretation is ultimately ruled out. In several reading experiments, Kaschak and Glenberg find that repeated exposure to the temporarily ambiguous non-standard structure, compared to repeated exposure to the unambiguous standard *needs to be*+participle structure in (5), facilitates later processing of both the non-standard *needs* structure (4a) and the modifier structure (4b), in line with the episodic processing account.

1. The meal needs to be cooked given that dinner is in an hour. (standard *needs*)

What does the episodic processing account say about the results reported here? Consider a temporarily ambiguous sentence of the kind employed in the experiments reported above.

1. The experienced soldiers warned about the dangers conducted the midnight raid.

The episodic processing proposal advanced by Kaschak and Glenberg predicts that, in sentences such as (6), subjects will initially retrieve the MV representation at the verb *warned*, since there is a stronger *a priori* expectation for this structure than for the RC structure, and, crucially, this retrieval should subsequently *facilitate* processing of that structure, even though the MV interpretation is ultimately ruled out. This prediction is not supported by either of our experiments. On the contrary, Experiment 2 finds that repeated exposure to RCs leads to *slower* processing of MVs (as predicted by the hypothesis advanced here).

How can we reconcile the apparently discrepant findings reported in this paper on the one hand and in Kaschak and Glenberg on the other? One caveat to comparing the results of Kaschak and Glenberg to the current results is that the experiments reported by Kaschak and Glenberg concern adaptation to novel–i.e., previously ungrammatical–syntactic constructions, whereas the experiments reported here utilized fully grammatical constructions that differed only in the extent to which subjects initially expected them. As a result, the materials used by Kaschak and Glenberg (particularly sentences like (4a)) were likely to be particularly jarring for subjects. It seems plausible that seeing a structure that is so initially striking may lead subjects to actually reflect on what they have just read and compare the novel structure (4a) to the one they expected (4b). Such materials may therefore more strongly engage episodic memory and lead to the types of effects observed by Kaschak and Glenberg. In short, future work is required to determine whether episodic processing and incremental adaptation to the statistics of the environment are in fact mutually exclusive, or whether these two mechanisms are jointly operative, with behavioral data more obviously consistent with one proposal versus another depending on the specific experimental materials.

### Syntactic Adaptation and Statistical Learning

In a line of research that has proceeded largely independently of research on syntactic priming, the statistical learning literature has generated a great deal of evidence that language users possess the ability to quickly learn distributional regularities over linguistic units from artificial languages such as syllables, words, and syntactic categories [63]–[65], [81], [82]. For instance, Saffran and colleagues showed that both children and adults exploit transitional probabilities between syllables in order to segment words in the input. Similarly, numerous researchers using artificial language learning paradigms have found evidence that knowledge of syntax might be acquired on the basis of statistical regularities defined over syntactic and lexical categories [81]–[83]. These results demonstrate that adults maintain a remarkable capacity for learning statistical regularities when *acquiring* the lexicon or grammar of a language. But do adults actively engage in a qualitatively similar sort of “statistical learning” during comprehension of their native language? In other words, are the same or similar learning processes at play during language acquisition also active throughout adulthood? The experiments reported here suggest that the answer may be yes. Moreover, ongoing work seeks to directly test this hypothesis by asking whether the same cortical areas implicated in visual and auditory statistical learning tasks [84]–[86] are also implicated in syntactic adaptation tasks such as those described in this paper.

Finally, a small number of recent studies directly address the notion that there is a link between online sentence comprehension and statistical learning. These studies build on experience-based models of language processing, discussed above. The experience-based processing studies cited above all employed estimates of syntactic expectations based on norming studies that averaged across many subjects or based on language corpora (which also implicitly average across speakers). While tests based on such estimates of average experience constitute an important step in establishing experience-based accounts, stronger evidence for experience-based accounts, and tentative support for our claim, comes from the finding that *individual differences* in language experience are reflected in expectation-based processing difficulty [28], [46], [87], [88].

MacDonald and Christiansen (2002) [46], for example, present simulations using simple recurrent networks (SRNs; [24]) to evaluate an experience-based interpretation of previously observed individual differences in language comprehension. Specifically, they wished to revisit previous claims that individual differences in language comprehension reflected variability in working memory span across individuals. For example, King and Just (1991) [89] found that differences in the comprehension of syntactically complex sentences such as object-extracted relative clauses (e.g., *The reporter the senator attacked admitted the error*) correlated with working memory span, such that high-span readers exhibited less difficulty comprehending these structures than low-span readers. MacDonald and Christiansen (2002) [46] trained the networks on a corpus of sentences from a context-free grammar whose statistical properties closely resembled corpora of English. In the context of the current discussion, the crucial result from their study was that, when the networks were tested on object-extracted relative clauses, network performance matched the performance, reported by King and Just, of the low-span readers after one epoch of training, but gradually came to resemble the performance of high-span readers after a second and third epoch. Based on these and other findings, MacDonald and Christiansen argue that language processing skills likely reflect an interaction of the cognitive architectures underlying language understanding as well as the specific linguistic experiences of individuals.

A more direct test of the hypothesis that linguistic expectations are shaped by individual, idiosyncratic linguistic experience is presented by Wells et al. (2009) [28]. In their groundbreaking study, subjects visited the lab multiple times and were exposed to a large number of object-extracted relative clauses. Wells and her colleagues found that repeated exposure over several days significantly diminished the processing difficulty normally associated with the object-extracted relative clauses.

The simulations reported by MacDonald and Christiansen and the human data reported by Wells et al. provide evidence for a relationship between linguistic experience and online language comprehension. But they do not directly test the claim that the effect of experience on language comprehension is mediated by statistical learning. Addressing this, Misyak and Christiansen (2012) [27] investigate the relationship between statistical learning, language experience, online language comprehension (of a variety of structures, including relative clauses), and a number of other individual differences variables previously cited in the literature. They find that online language comprehension performance is best explained in terms of subjects’ performance on a separate statistical learning task.

As mentioned in the introduction, however, previous work on experience-based processing, syntactic priming, and statistical learning has all proceeded largely in parallel, and has left open the question of how the immediate effect of experience on language comprehension can accumulate over time to give rise to cumulative priming, experience-based processing effects, and environment-specific adaptation. We have attempted to synthesize these different lines of work by demonstrating that syntactic adaptation can be profitably construed as the rapid, incremental, and cumulative convergence towards the statistics of a novel linguistic environment. Next, we further explore the related question of whether the adaptation effects we observed are mediated by a learning mechanism.

### What Mechanisms Underlie Syntactic Expectation Adaptation?

A great deal of previous work on syntactic priming concerns the question of what *mechanism* gives rise to syntactic priming. Two main competing views have emerged out of this line of research. Transient activation accounts hold that priming results from a short-lived boost in the activation of a syntactic representation [72], [90]. By contrast, implicit learning accounts hold that priming is a consequence of an implicit learning mechanism [23], [91], [92]. We believe that implicit learning accounts (in particular, [23]) cover the current results most naturally. Before elaborating on this claim, we briefly review previous findings that have been argued to distinguish between implicit learning and activation-boost accounts.

At least three types of results–almost exclusively from work on language production–have been argued to adjudicate between transient activation and implicit learning accounts of priming, and there is as yet no consensus. First, some researchers have proposed that implicit learning accounts, but not transient activation accounts, predict long-lasting syntactic priming effects [91]. Research on this question has found conflicting results, with some researchers finding long-lasting syntactic priming [28], [91], [93] and others rapidly decaying syntactic priming [90] (for recent attempts to reconcile discrepant findings see [94], [95]). A second, related argument holds that only implicit learning accounts predict cumulative priming since effects of implicit learning should persist beyond the most recent syntactic prime [92], [96]. Such cumulativity has indeed been observed (for production, see [92], [96]; for comprehension, see the current results as well as [59]).

One challenge with using the longevity of priming effects as a way of distinguishing competing accounts is that it is unclear what “long-lasting” means [97], [98]. Most previous studies seem to interpret “short-lived” as “limited to the most recent prime” [90] or “rapidly (i.e., logarithmically or power-law) decaying” [95], whereas effects persisting over several sentences have been interpreted as “long-lived” [91], [92], [96]. While there seems to be some agreement in the literature that even persistence of priming effects without measurable decay over a few sentences favors an implicit learning account of syntactic priming, it is possible that “short-term” activation boosts could also account for such effects. Only very recently, a handful of studies have examined and found syntactic adaptation over multiple days [28], [99]. At this point it is an open question whether such long-term adaptation is due to the same mechanisms as rapid syntactic adaptation as investigated here (including what has commonly been called syntactic priming). If so, this would arguably be a strong argument in favor of implicit learning accounts of syntactic priming.

A third argument that has been brought forward in favor of implicit learning accounts is the observation that the strength of syntactic priming seems to be sensitive to the prediction error associated with the syntactic prime (for production see [23], [26], [100]; for comprehension, see [25]). This prediction follows naturally from error-driven implicit learning accounts of syntactic priming [23], [101] (for alternative supervised learning accounts in a Bayesian framework, see [16], [17]). Reitter et al. (2011) [95] show that unsupervised implicit learning can account for some of the observed sensitivity of syntactic priming to the prime’s prediction error. We refer to Fine and Jaeger (2013) [25] and Jaeger and Snider (2013) [26] for further discussion and data that challenges the account advanced in Reitter et al. Regardless of the outcome of this discussion, there is currently no account that explains the apparent sensitivity to the prime’s prediction error without referring to some form of implicit learning.

Situated within these arguments, our results support an implicit learning account of priming. Although we do not test the longevity of the adaptation effects observed in our experiments, in both experiments subjects are sensitive to the cumulative statistics of the environment. That is, the degree to which subjects’ expectations for a structure have changed at a given point in the experiment depends on how many times subjects have seen (a) that structure and (b) other structures competing for probability mass. To the extent that transient activation accounts do not predict cumulative priming and insofar as learning accounts do, our results appear to support an implicit learning account.

Second, the adaptation effects we observe provide indirect evidence for error-sensitivity [23], [25], [26]. That is, we observed changes in RTs over the course of both experiments for both RC and MV structures. However, the magnitude of the change in RTs was greater for RCs than for MVs. A slightly different way of saying the same thing is that observing a low-probability linguistic event (and therefore one with a relatively large error signal) leads to greater changes in reading times (especially if the error signal is proportional in the surprisal, rather than probability or relative frequency; for further discussion, see [26]).

We believe there are significant advantages to adopting an implicit learning account of syntactic adaptation (and hence, by extension, of syntactic priming)–and specifically, the hypothesis that adaptation serves to facilitate efficient language processing by adjusting linguistic expectations to linguistic environments. Perhaps most promisingly, this approach holds the potential to free syntactic priming from its status as a phenomenon that is frequently studied in a manner divorced from recent experimental and computational work on adaptation and learning in other cognitive domains (see next section, where we also discuss exceptions to this trend).

### Adaptation as a Domain-General Principle

Moving beyond specific debates surrounding adaptation in language processing, previous work on adaptation from numerous research traditions in perception and cognition suggests that adaptation is a fundamental property of the human brain. From single neurons to observable action, behavior is to a large degree a matter of responding and adapting to experience at multiple timescales. In a recent paper, Clark (2012) [102] discusses the central role of experience-driven changes in behavior, reviewing more than a century of work in perception, action, cognition, and neuroscience leading to the insight that the brain is fundamentally a “prediction machine”, and that the purpose of the brain can be profitably construed as modifying perception and behavior in order to reduce error signals, i.e., the difference between what is expected and what is observed [103], [104]. In vision, for example, humans have been shown to adjust their representation of the relationship between visual cues (e.g., depth and blur) according to recent experience [105] (for further work on adaptation in vision, see [106]–[110]). In motor control, humans rapidly adapt reaching movements in response to experimentally controlled perturbations in motor feedback [111]–[113]. Experience, prediction, and adaptation play similarly crucial roles in describing spatial categorization [114] and in higher-level cognitive phenomena such as face and object recognition [114]–[116].

Focusing on language in particular, the last few years have seen a considerable amount of work framed in terms of adaptation. This work comes from research on phonetics [117]–[119], lexical processing [120], [121], prosody [122], pragmatics [123], and syntax (e.g., the current study as well as [17], [26], [58], [60]). But despite work on adaptation across linguistic domains, and explicit suggestions by some language researchers that lifelong learning (adaptation) is a general property of the systems underlying language processing [23], [24], [46], [48], [72], [124], researchers across linguistic domains have often explored these questions in isolation from each other, and in isolation from research on adaptation outside of language processing. However, there are qualitative and quantitative parallels emerging in work on adaptation across linguistic domains. For example, the literature on phonetic adaptation has centered largely on a cluster of related phenomena whereby listeners adjust their representations of the boundaries of phonetic categories (i.e., of what percepts count as belonging to one phonetic category or another, e.g., [117]). Syntactic and phonetic adaptation are similar insofar as, in both cases, humans seem to use often environment-specific statistical information in the linguistic signal in order to inform the inferences and predictions they make about intended messages. Lending further support to the analogy between phonetic and syntactic adaptation, the adaptation effects observed here arise at a similar timescale as those observed in phonetic adaptation (i.e., within only a handful of sentences; cf. [125], [126]), suggesting a quantitative as well as a qualitative parallel. To the extent that evidence from adaptation in other linguistic processes, such as pragmatic and prosodic processing, exists (see above), this too seems to exhibit a similarly rapid time course [122], [123].

To the extent that we have a vocabulary in place for discussing adaptation across linguistic domains, it may not be entirely premature to go further and ask whether adaptation of the kind observed in language experiments shares any commonalities with adaptation effects observed in non-linguistic domains. For example, recent and ongoing computational work may provide additional leverage in bridging investigations of adaptation in language with adaptation in other domains. For instance, recent modeling work on syntactic adaptation [16], [17] and phonetic adaptation [127]–[131] has successfully employed the same computational framework previously applied to adaptation in non-linguistic domains (Bayesian inference and belief-updating, e.g., [111], [112], [132] ). While it remains to be seen whether a unified, domain-general computational treatment of adaptation is possible, this recent work at least points to shared computational principles and the potential parsimony and insight that can be achieved by thinking of these phenomena in similar terms [102], [104], [133]–[135].

### Methodological Consequences

Finally, our findings, together with previous work on syntactic priming, pose a methodological question for behavioral work on language processing: if humans continuously adapt their expectations to the statistics of the linguistic environment, then adaptation should be operative in *any* behavioral experiment, and should become more pronounced as the statistics of the experiment differ more from subjects’ prior experience (see Jaeger [136], p. 52–53 for discussion of this point).

What does this mean for experiments on syntactic processing and the interpretation of previous experiments? The current study suggests that the effect adaptation exerts on behavior does not completely obscure the effect of other variables of interest: for both experiments reported above, we replicated findings from previous work concerning the effects of temporary syntactic ambiguity [41] and *also* found evidence for adaptation. At least two points follow. First, to the extent that subjects are adapting to the statistics of language processing experiments, it is likely that the magnitude of certain types of effects are being systematically *misestimated*. For instance, in Experiment 1 we replicate the classic ‘garden path effect’ in sentences temporarily ambiguous between the MV and RC reading. However, the magnitude of this effect changes substantially throughout the experiment. Previous research on the RC/MV ambiguity thus likely underestimated the true effect size of the garden path. One consequence of this is that researchers risk null or even reversed effects of prior expectations if the statistics of the experiment deviate strongly from those prior expectations. Ironically, this risk *in*creases as the number of critical items in the experiment increases. This problem is not a hypothetical one, as recent debates show (for an example of how failure to account for expectation adaptation within a psycholinguistic experiment can fundamentally alter the interpretation of behavioral results, see the recent exchange in [60], [137], discussed above).

### Future Directions

We have provided evidence for the claim that comprehenders rapidly adapt to the statistics of novel linguistic situations. The work reported here leaves a number of fundamental questions unresolved. Some outstanding issues and topics for further work were mentioned above. Here, we discuss some additional avenues for further work that we find particularly compelling.

One major question is, assuming there is rapid adaptation to the statistics of the environment, what subsequently happens to this knowledge? Is this knowledge maintained and, if so, is it generalized and brought to bear on subsequent linguistic situations? Kamide (2012) [58], discussed above, offers compelling evidence that, at a minimum, comprehenders’ adapted syntactic expectations can be indexed to specific talkers. Kamide’s results thus suggest that the results of syntactic adaptation can be maintained insofar as comprehenders maintain separate subjective statistics for multiple talkers in a given environment (put another way, talkers condition their subjective estimates of the probabilities of various structures on talker identity).

This raises the question of how long subjects’ adapted expectations are maintained. There are some indications in the literature that the effects of experience on language comprehension may be rather long-lasting. For example, Wells et al. (2009) [28] find evidence that exposure to distributions of linguistic materials results in adaptation effects that persist at least for several days. These findings are reminiscent of evidence from both word identification [138] and phonetic adaptation [139] tasks. Directly probing the longevity of the effects observed here and the circumstances that promote and diminish longevity is a topic for ongoing and future work.

Assuming that comprehenders can indeed maintain what they learn during adaptation, how is this knowledge generalized and integrated with subsequent and novel linguistic environments? For example, in a paradigm like that used here, to what extent would subjects generalize what they learn to a new experimental context (e.g., to a different room)? Similarly, how would subjects in the Kamide (2012) [58] study generalize what they learned about the various talkers in that experiment to subsequently observed talkers? Our experiments in fact raise a related question concerning generalization relevant also for the literature on syntactic priming: do syntactic adaptation effects of the kind observed here depend on verb repetition (or “lexical overlap”, e.g., [20])? Another way of putting the question is, do subjects track the statistics of syntactic structures, averaging across experience with all verbs, or do they track verb-specific statistics? Previous work on syntactic priming is equivocal on the matter of whether lexical overlap is [22] or is not [20], [21] required to observe syntactic priming. The results of our first experiment at least tentatively suggest that lexical overlap is not required (cf. analysis on p. 20); the materials of our second experiment featured lexical overlap and the results from that experiment therefore do not directly bare on the matter of whether lexical overlap is *required*. We suspect a fruitful path forward will be to consider the possibility that adaptation is not either lexically independent or not, but rather that subjects make generalizations to new verbs in certain situations in not others (e.g., when the verbs used in a particular study are sufficiently similar along some dimension to which the subjects are sensitive; for further discussion, see Jaeger and Snider [26], p. 74). Fleshing out this approach to the question of lexical overlap in syntactic priming remains a topic for ongoing work.

It is also likely that certain social variables are relevant to adaptation. For example, are the types of generalizations made by comprehenders constrained by beliefs about the social variables that might give rise to differences in the way people use language [140]? This work could profitably build on a small body of previous work in phonetic adaptation that has explored the circumstances under which phonetic adaptation is observed and when adaptation is generalized to new talkers or new speech sounds [118], [125]. We believe similar work on syntactic adaptation will allow us to begin to further articulate the relative contributions of learning, episodic memory, and social cognition to language processing and adaptation.

## Conclusions

We have found that comprehenders continuously adapt their syntactic expectations to the statistics of novel linguistic environments and that the resulting environment-specific expectations can overturn expectations based on previous experience. To the extent that adaptation of the kind observed here represents a form of implicit learning, our findings suggest that the mechanisms described in the statistical learning literature may be operative not only in the acquisition of new languages, but also in the continuous maintenance and adjustment of linguistic expectations throughout adulthood. Moreover, our findings extend previous work on syntactic priming, and we have suggested that findings previously discussed under the heading of syntactic priming may be manifestations of a very continuous set of processes and mechanisms common to multiple levels of language processing as well as non-linguistic domains. Following work in these areas, we argue that adaptation at the level of syntax serves the function of maximizing the efficiency with which syntactic information is processed, because inferences about syntactic structure during online language understanding are only helpful given sufficiently accurate beliefs about environment-specific syntactic statistics. Taken together with other work on syntactic and pragmatic processing, our results therefore suggest that adaptation is likely to be a general property of language processing, just as has been observed across other domains of human cognition, and an essential ingredient in the ability of humans to cope with a dynamic environment.

## Supporting Information

Appendix S1 **Additional information on the analysis of Experiment 1.**
(DOCX)

Appendix S2 **Materials used in Experiment 2.**
(DOCX)
