# Supplementary material for: Rapid Expectation Adaptation during Syntactic Comprehension
Source: PLoS One. 2013 Oct 30;8(10):e77661. doi: 10.1371/journal.pone.0077661 (PMC3813674; doi:10.1371/journal.pone.0077661)
Supplement: Appendix S1 — Additional information on the analysis of Experiment 1. (DOCX) [file pone.0077661.s001.docx]

**Appendix S1: Additional information on the analysis of Experiment 1**

**Fixed effects**

Fixed effects for the model reported in Experiment 1 included the full factorial design (i.e., all main effects and higher-order interactions) of sentence type (main verb vs. reduced relative), ambiguity (ambiguous vs. unambiguous), and item order, an index from 1 − 36 of when in the experiment the subject saw each item, relative to other items. Additionally, the model included a main effect of log stimulus order, which is an index of when in the experiment a subject saw an item, relative to both items and ﬁllers. Including this main effect is intended to control for “task adaptation”. We log-transformed this predictor because previous work in our lab has demonstrated that this transformation achieves a slightly better ﬁt [1–3], which also holds for the current data. All main effects were centered at zero to reduce collinearity between higher-order interactions.

**Random effects**

To determine the appropriate random effects structure for the model, we followed the procedure described in [4] (see also http://hlplab.wordpress.com/2011/06/25/more-on-random-slopes/). We performed a forward step-wise model comparison procedure in which we iteratively added random effects to the model and compared each resulting model to the previous one. Models were compared using log-likelihood ratios. To do this in R, we used the *anova()* function, e.g., *anova(modelA, modelB)*. All models started with only random intercepts for subject and item. We provide pseudo R code to illustrate:

Model1 <- RT ~ SentenceType * Ambiguity * ItemOrder + (1|Subject) + (1|Item)

Next, we add a by-subject random slope for SentenceType:

Model2 <- RT ~ SentenceType * Ambiguity * ItemOrder + (1+ SentenceType|Subject) + (1|Item)

We then compare these models using the *anova()* function:

anova(Model1, Model2)

If the result of this test is signiﬁcant, then the by-subject random slope for SentenceType is justiﬁed by the data based on model comparison. We therefore leave it in the model and add a by-subject random slope for ambiguity:

Model3 <- RT ~ SentenceType * Ambiguity * ItemOrder + (1+ SentenceType+Ambiguity|Subject) + (1|Item)

We then compare Model2 and Model3 using the anova function. If the result is signiﬁcant, this random slope stays in the model and we then test for a by-subject random slope for the SentenceType by Ambiguity interaction; if not, it is taken out.

We iterate through all the possible by-subject and by-item slopes this way. The model resulting from this procedure is given above in the main text. For Experiment 1, the random effects structure included random intercepts for subject and item, as well as by-subject random slopes for ambiguity and item order, as well as by-item random slopes for sentence type, ambiguity, and item order. The results reported for both experiments also hold if the full random effects structure is used (as advocated by [5]). Note that the procedure used here was not tested by Barr et al., and has not been shown to lead to increased Type I error rates.

**References**

1. Fine AB, Qian T, Jaeger TF, Jacobs RA (2010) Syntactic Adaptation in Language Comprehension. Proceedings of the 2010 Workshop on Cognitive Modeling and Computational Linguistics. Uppsala, Sweden: Association for Computational Linguistics. pp. 18–26.

2. Hofmeister P, Jaeger TF, Arnon I, Sag IA, Snider N (2011) The source ambiguity problem: distinguishing the effects of grammar and processing on acceptability judgments. Language and Cognitive Processes.

3. Jaeger TF (2007) Using Corpora for Research on Language Production. International Conference on Processing Head-final Structures. Rochester, NY.

4. Jaeger TF (2009) Random effect: Should I stay or should I go? Human Language Processing lab blog. Available: http://hlplab.wordpress.com/2009/05/14/random-effect-structure/. Accessed 16 September 2013.

5. Barr DJ, Levy R, Scheepers C, Tily HJ (2013) Random effects structure in mixed-effects models : Keep it maximal. Journal of Memory and Language 68: 255–278.
