# Supplementary material for: Rapid Expectation Adaptation during Syntactic Comprehension
Source: PLoS One. 2013 Oct 30;8(10):e77661. doi: 10.1371/journal.pone.0077661 (PMC3813674; doi:10.1371/journal.pone.0077661)
Supplement: Appendix S2 — Materials used in Experiment 2. (DOCX) [file pone.0077661.s002.docx]

**Appendix S2: Materials used in Experiment 2**

**Block 1 Items {ambiguous, unambiguous} for RC-First group**

1. The kitchen staff {who were fed, served} in the cafeteria got very sleepy.
2. The young technician {taught, who was shown} the computer program caught on right away.
3. The young children {watched, who were seen} in the hallway were following the adults.
4. The thoughtless secretaries {called, who were reprimanded} on the balcony returned to their desks.
5. The silly boys {called, who were reprimanded} during the play quickly left the auditorium.
6. Several angry workers {warned, who were told} about low wages decided to file complaints.
7. The calico cat {washed, who was bathed} in the alley ran into the street.
8. The frightened kid {pushed, who was shoved} through the crowd got separated from Jane.
9. A yellow frisbee {dropped, that was thrown} from the roof landed in the ditch.
10. An impatient shopper {pushed, who was shoved} through the doors complained to the manager.
11. The experienced soldiers {warned, who were told} about the dangers conducted the midnight raid.
12. The sick child {washed, who was bathed} early every morning wanted her rubber duck.
13. The six volunteers {taught, who were shown} the complicated procedure became very good students.
14. The evil genie {served, who was fed} the golden figs went into a trance.
15. The large package {dropped, that was thrown} from the plane hit several tall trees.
16. The brown sparrow {watched, who was seen} on a branch pecked at an insect.

**Block 1 Fillers for Filler-First Group**

1. Each of the divers had a strict regimen so that they would be prepared for the swim meet.
2. Many of the soldiers were looking forward to going home for the winter holidays.
3. The people downtown are frustrated by the lack of available parking.
4. The leader of the gambling ring was always mistrustful of his bodyguards.
5. The university students sometimes move into the dormitories as early as August.
6. The runners were in much better shape in the fall than in the winter.
7. The parents completely disagreed with the new regulations.
8. The wealthy bankers liked to frequent the bars downtown.
9. The roofer got a terrible sunburn from being outside all day.
10. The priceless ceramic sculpture had to sit on the top shelf of the lawyer's office.
11. The university's math courses were among the nation's most rigorous.
12. The company's health plan did not cover even the most basic health services.
13. Cameron's French class went on a trip to Paris one summer to improve their language skills.
14. Max's wedding had to be rescheduled because of a hurricane.
15. The unpopular anthropology professor was finally going to retire.
16. The cyclist wanted to train throughout the winter so he moved to Hawaii.

**Block 2 Items {ambiguous, unambiguous} (in bold) and fillers for both groups (in the actual order used in the experiment)**

1. The term papers from the previous semester were beginning to accumulate on the teacher’s desk.
2. The shoppers love to spend all day at the mall on the weekends.
3. **The eager bartender {served, who was fed} the fried snacks worked till past midnight.**
4. The coffee shop was a popular hangout for political activists.
5. **The experienced chef {warned, who was told} about the stove burnt his hand anyway.**
6. The school principal had to work constantly all summer dealing with paperwork.
7. The lawnmower did not always function properly when the grass was wet.
8. The new student disappeared after only three days of school.
9. **The dirty hikers {washed, who were bathed} in the stream set up their camp.**
10. The storekeepers were afriaid that riots would ensue after the hometeam won the tournament.
11. The real estate agent blundered when he revealed the house's plumbing problems.
12. The library was open to all members of the community since it was supported by tax dollars.
13. **The teenage girls {called, who were reprimanded} in the hallway answered the principal rudely.**
14. The physics professor at the university was finally going to retire.
15. The landscaper boasted of his achievements constantly.
16. The dance troupe came to set up their equipment.
17. **Some rescue workers {warned, who were told} about the avalanche decided to stand by.**
18. The chess match lasted for hours and finally ended in a stale mate.
19. The street lamps usually came on automatically just before dark.
20. **The Indian children {washed, who were bathed} in the stream splashed and shouted loudly.**
21. The foreign ambassadors arrived to the meeting surrounded by security guards.
22. The sunburned boys who were fed the hot dogs got a stomach ache.
23. Many of the city cops refuse to work in the rough parts of town.
24. The car salesman waited anxiously for more customers.
25. **The nervous wrestler {pushed, who was shoved} through the crowd hadn't seen his opponent.**
26. All the guitarists learned to play when they were teenagers.
27. **A small dog {pushed, who was shoved} through the fence hurt his hind legs.**
28. The angry customers decided to leave the restaurant.
29. **The computer programmers {called, who were reprimanded} about the problem knew what to do.**
30. The girls on the basketball team tried to practice all summer.

**Block 3 Items {ambiguous, unambiguous} (in bold) and fillers for both groups (in the actual order used in the experiment)**

1. The children in the park could be heard three blocks away.
2. **The hippie children {washed, cried} in the river while their parents fished.**
3. The power plant deserved more attention from the candidates during the election.
4. The quilts were sold by the side of the road for ten dollars.
5. **The drunk tourists {called, giggled} on their balcony as cars drove by.**
6. The pilots flew over the city where they had just had a wonderful weekend.
7. **The experienced waitress {served, ate} the grilled chicken in the nice restaurant.**
8. The prisoners were unable to cross the field without being seen.
9. **The sleepy volunteers {served, ate} the hot soup in the aid station.**
10. Each of the cab drivers had their own favorite route to get to the airport.
11. **The aging professors {warned, spoke} about the midterm just before fall break.**
12. The new student caught everyone's attention when he came into the room.
13. **The hospital patient {washed, cried} in his room after eating his breakfast.**
14. The valuable lamp was broken by the mischievous boy.
15. All the undergraduates in the class had trouble keeping up.
16. **The overprotective mother {pushed, went} into the office to berate the principal.**
17. The package arrived too late to be of any use.
18. **The grad students {called, giggled} during their exams until the professors saw.**
19. The engineers at the plant had to wear helmets when they went near the machines.
20. **The pawnshop owner {pushed, went} through the doors to his storage room.**
21. The new experiment was the source of a great deal of excitement in the lab.
22. **The cotton farmers {warned, told} about bad floods just before harvest time.**
23. The eccentric professor always inspired his students to think critically about their work.
24. The former drug addict's memoirs were met with critical acclaim.
25. The laptops were too expensive for most of the students.
